# Malignant Range Elevation of Serum Chromogranin A due to Inadvertent Use of Proton Pump Inhibitor in a Subject with Pancreatic Incidentaloma

**DOI:** 10.1155/2011/342480

**Published:** 2011-07-10

**Authors:** Usman Hammawa Malabu, Rozemary Karamatic, Gillian Mahy, Kunwarjit Singh Sangla

**Affiliations:** ^1^Department of Endocrinology, Townsville Hospital, 100 Angus Smith Drive, Douglas QLD 4814, Australia; ^2^Department of Gastroenterology, The Townsville Hospital, 100 Angus Smith Drive, Douglas QLD 4814, Australia

## Abstract

We present a case of highly elevated tenfold rise of serum chromogranin A in a young, morbidly obese, hypertensive female being investigated for pancreatic mass, weight loss, and elevated ESR. Following extensive noninvasive investigations, an ultrasound-guided pancreatic biopsy confirmed benign haemorrhagic cyst. A clue to the etiology of the hyperchromogranin A was the elevated serum gastrin level leading to suspicion of proton pump inhibitor administration confirmed by admittance to its use. Withdrawal of the medication led to dramatic resolution of the neuroendocrine tumor marker.

## 1. Introduction

Chromogranin A (CgA) has shown to be a useful marker in the diagnosis and followup of neuroendocrine tumors (NETs). Its reliability in correctly diagnosing NETs is a growing concern [1]. Proton pump inhibitors (PPIs) being widely used for treating upper gastrointestinal disorders including gastroesophageal reflux disease (GORD) may cause serious differential diagnostic problems with elevation of serum CgA in suspected NETs [2]. As far as we know, there is no report of this artefact causing diagnostic interference in subjects with pancreatic mass requiring further characterization. We report a case of highly elevated serum CgA in a patient with pancreatic incidentaloma caused by PPI therapy.

## 2. Case Presentation

**Figure 1:**
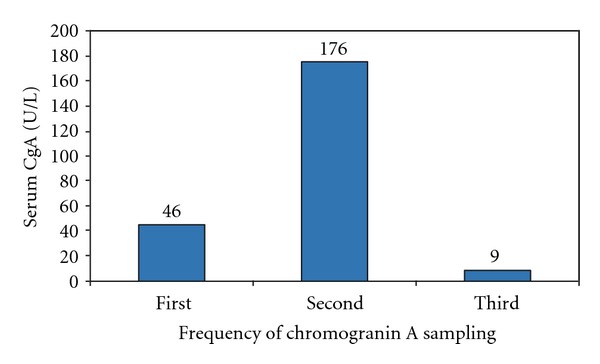
Marked escalation of serum chromogranin A levels 4 months after initial sampling on proton pump inhibitor rapid normalisation after 3 weeks of its withdrawal.

**Figure 2:**
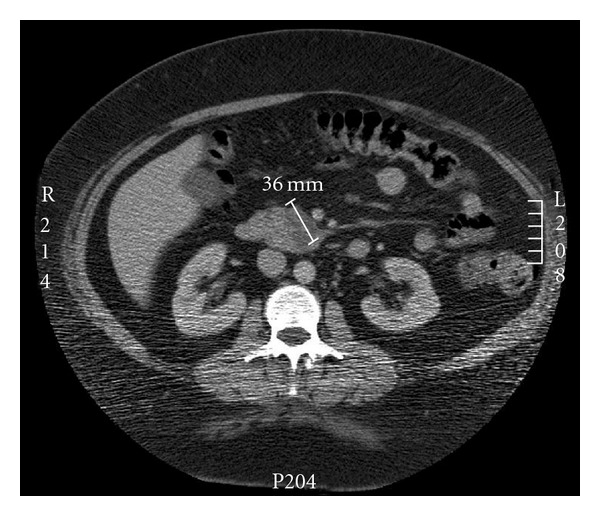
CT scan of the abdomen showing a 3.6 × 2.4 cm cystic lesion within the uncinate process of the pancreas extending into the pancreatic head.

A 46-year-old morbidly obese Caucasian female was referred by her general practitioner (GP) to rheumatologist for further evaluation of 25 kilograms weight loss over a period of one year associated with persistently elevated ESR of 60 mm/hr. Her history was remarkable for hypertension, depression, and GORD. Medications disclosed at initial consultation included verapamil SR 240 mg/day for a well-controlled hypertension. Clinical examination at presentation revealed an obese woman with a body mass index of 58 kg/m^2^ without clinical features of Cushing's syndrome. The rest of the physical examination was normal. All rheumatologic and vasculitic studies were unremarkable. As part of the work-up, an ultrasound and CT scan showed pancreatic head mass measured 3.6 × 2.4 cm diameter. The radiological differentials were cystoadenoma and adenocarcinoma. Followup liver function test showed no evidence of biliary obstruction. Joint gastroenterological and endocrine biochemical assessment revealed normal serum glucose, glucagons, and vasoactive intestinal peptide. Other tumor markers were undetectable. Further investigations included normal findings for cortisol rhythm and low dose dexamethasone suppression. Urinary catecholamine and 5-hydroxy indole acetic acid excretion did not suggest elevated hormonal activity. An initial serum CgA measured by enzyme-linked immunosorbent assay (0.5 U/L detection limit; Dako, Denmark) was noted to be moderately elevated 46.0 U/L (normal < 17.2 U/L) and rose to 176 U/L in 4 months (Figures 1 and 2). Simultaneous serum gastrin level was elevated twice upper limit of normal 198 ng/L (normal < 100 ng/L). Further clinical review highlighted previously undisclosed usage of PPI, rabeprazole 40 mg once daily dating back to the twelve-month period of work-up. This medication was then subsequently suspended resulting in normalization of CgA (Figure 2) and subsequent 6-month followup confirmed consistently undetectable serum CgA levels while off the PPI. 131I-MIBG scintigraphy did not show pathologic isotope accumulation and serial CT scan of abdomen revealed no increase in size of the pancreatic mass. Endoscopic ultrasound-guided fine needle biopsy confirmed the lesion to be a benign pancreatic hemorrhagic cyst.

## 3. Discussion

We have demonstrated another evidence for dramatic rise and fall of CgA in a patient who had been investigated for marked weight loss and elevated ESR. Other biochemical profiles were unremarkable, yet, elevation of the tumor marker in the malignant range eventuated in significant anxiety for both patient and doctors. It was not known to us that the patient had been on PPI introduced during the period of work-up by her GP for GORD. This was complicated by an apparent weight loss possibly from anorexia due to exacerbation of depression symptoms. The serum ESR remained high throughout with no cause identified during the course of investigation. While rising level of CgA is not new in the literature [1, 2], the progressive rise of CgA in association with symptoms particularly in an individual with pancreatic mass was an interesting aspect of our report. Igaz et al. reported a 7-fold rise of CgA due to PPI which was normalized upon stoppage of the medication [3]. To our knowledge, this is the first report of a very high level of serum CgA of 10-fold magnitude due to ingestion of PPI. 

Another interesting phenomenon highlighted by this case is the report of rapid normalization of CgA after withdrawal of PPI in a subject presenting with a pancreatic non-functioning incidentaloma. Similar findings were reported in subjects with bilateral adrenal incidentaloma in which persistently elevated CgA after adrenalectomy for phaeochromocytoma was normalized only following suspension of PPI [3]. Our report as well as others observations serve as a reminder to clinicians to pay great attention to confounding factors before pursuing invasive and costly procedures in suspected cases of NETs. The fact that the serum gastrin level was elevated in our patient pointed to a possible usage of PPI which was confirmed on reviewing her medication history. 

The pathogenesis of increased levels of CgA by PPI is not clear. For instance, although hypergastrinemia induced release of the tumor marker was hypothesized as a possible explanation [4, 5], Gori et al. in fact reported lack of concomitant elevation of serum gastrin levels associated with the use of PPI [6]. We measured this peptide simultaneous with CgA and it was modestly elevated in keeping with others findings [4, 5]. Furthermore, other nonmalignant endocrine conditions leading to hypergastrinemia were reported to cause increased serum CgA [7]. Interestingly, recent reports have suggested that chronic use and dose escalation of PPI correlates with increased level of both CgA and serum gastrin [3, 5]. Our patient was maintained on the same dose of PPI throughout the period of use yet; in the last 4 months there was an alarmingly marked elevation of CgA. Another possible reason for this might be poor preparation for the CgA sampling such as nonfasting state [8]. Other confounding factors including use of non-PPI medications such as glucocorticoids were not contributory. On the other hand, the period from withdrawal of PPI to subsequent CgA decline was suggested in a recent report to be one week [3] while others earlier proposed 7–10 days [2]. In our subject, the decisive fasting serum CgA was analysed after complete suspension of the PPI for a period of 3 weeks which led to dramatic normalization of the tumor marker. 

This case suggests that despite the potentially complex pathophysiologic and biochemical pathways resulting in increased CgA associated with a pancreatic mass in the context of PPI usage, prompt normalization of CgA levels was achieved upon PPI withdrawal. In order to save patients from invasive, risk prone, and costly investigations, it is crucial that guidelines are formulated that recommend that meticulous scrutiny of confounding factors is invoked. This should include comprehensive review of medications with particular attention to undisclosed acid-suppressant therapy [9]. This will certainly reduce the incidence of false positive diagnoses of NETs and avert unnecessary anxiety and investigations. We add this case to the growing list of reports of elevated CgA in neuroendocrine incidentaloma and for the first time report on the finding in a patient with a worrying pancreatic mass hoping to better define the clinical work-up of NETs and promote awareness to avoid unnecessary anxiety potentially relating to misdiagnosis.
